# Open versus laparoscopic left lateral hepatic sectionectomy within an enhanced recovery ERAS® programme (ORANGE II – Trial): study protocol for a randomised controlled trial

**DOI:** 10.1186/1745-6215-13-54

**Published:** 2012-05-06

**Authors:** Ronald M van Dam, Edgar M Wong-Lun-Hing, Gerard JP van Breukelen, Jan HMB Stoot, Joost R van der Vorst, Marc HA Bemelmans, Steven WM Olde Damink, Kristoffer Lassen, Cornelis HC Dejong, ORANGE II Study Group

**Affiliations:** 1 Department of Surgery, Maastricht University Medical Centre, Maastricht, The Netherlands; 2 Nutrim School for Nutrition, Toxicology and Metabolism, Maastricht University Medical Centre, Maastricht, The Netherlands; 3 Department of Methodology and Statistics, Maastricht University Medical Centre, Maastricht, The Netherlands; 4 Department of Surgery, Orbis Medical Centre, Sittard, The Netherlands; 5 Department of Surgery, Leiden University Medical Centre, Leiden, The Netherlands; 6 Department of Surgery, Royal Free Hospital, London, United Kingdom; 7 Department of Surgery, University of Northern Norway Hospital, Tromsö, Norway; 8 Clinical and Surgical Sciences, Department of Surgery, Maastricht University Medical Centre, PO Box 5800, 6202, AZ, Maastricht, The Netherlands

**Keywords:** Laparoscopy, Open liver resection, Hepatectomy, ERAS, Left lateral sectionectomy, RCT

## Abstract

**Background:**

The use of lLaparoscopic liver resection in terms of time to functional recovery, length of hospital stay (LOS), long-term abdominal wall hernias, costs and quality of life (QOL) has never been studied in a randomised controlled trial. Therefore, this is the subject of the international multicentre randomised controlled ORANGE II trial.

**Methods:**

Patients eligible for left lateral sectionectomy (LLS) of the liver will be recruited and randomised at the outpatient clinic. All randomised patients will undergo surgery in the setting of an ERAS programme. The experimental design produces two randomised arms (open and laparoscopic LLS) and a prospective registry. The prospective registry will be based on patients that cannot be randomised because of the explicit treatment preference of the patient or surgeon, or because of ineligibility (not meeting the in- and exclusion criteria) for randomisation in this trial. Therefore, all non-randomised patients undergoing LLS will be approached to participate in the prospective registry, thereby allowing acquisition of an uninterrupted prospective series of patients. The primary endpoint of the ORANGE II trial is time to functional recovery. Secondary endpoints are postoperative LOS, percentage readmission, (liver-specific) morbidity, QOL, body image and cosmetic result, hospital and societal costs over 1 year, and long-term incidence of incisional hernias. It will be assumed that in patients undergoing laparoscopic LLS, length of hospital stay can be reduced by two days. A sample size of 55 patients in each randomisation arm has been calculated to detect a 2-day reduction in LOS (90% power and α = 0.05 (two-tailed)).

The ORANGE II trial is a multicenter randomised controlled trial that will provide evidence on the merits of laparoscopic surgery in patients undergoing LLS within an enhanced recovery ERAS programme.

**Trial registration:**

ClinicalTrials.gov NCT00874224.

## Background

Liver resection for colorectal metastasis is the only potentially curative therapy, and has become the standard of care in appropriately staged patients, offering 5-year survival rates of approximately 35-40% [1]. For symptomatic benign lesions and those of uncertain nature or large size, liver resection is also a widely accepted treatment. Within the framework of optimising postoperative recovery and/or producing a shorter length of stay (LOS) in hospital, laparoscopic surgery and enhanced recovery programmes have recently been introduced for liver surgery.

Laparoscopic liver resection was first described in 1995 [2]. Over the past decade the method has gained wide acceptance for various liver resection procedures [3-9]. Multiple retrospective case series and reviews comparing open with laparoscopic liver resection indicate that laparoscopic liver resection can be used safely for both malignant and benign liver lesions [10-15]. Recent publications from expert centers show that a substantial part of the total volume of major and minor liver resections is performed laparoscopically, and results are good [16,17]. Laparoscopic liver resection is associated with shorter LOS, less postoperative pain, earlier recovery, and better quality of life (QOL) [9,13,18,19]. Comparing patients undergoing an open left lateral sectionectomy (LLS) of the liver with those undergoing laparoscopic LLS, both Vigano *et al*. and Carswell *et al*. [20,21] found no significant difference in operating time between the two groups. In addition, the median length of postoperative LOS was significantly less (6 vs. 9 days, *P* ≪ 0.01) after laparoscopic resection [3]. Furthermore, no evidence of a compromised oncologic clearance in laparoscopic liver resection has been found [3,13]. However, recovery and LOS are not only dependent on the type of surgery or procedure, and other variables should also be taken into account.

The Enhanced Recovery After Surgery (ERAS) programme has been introduced to improve postoperative care. This multimodal programme, derived from Kehlet’s pioneer work in the 1990s for multimodal surgical care, involves optimisation of several aspects of the perioperative management of patients undergoing major abdominal surgery. In patients undergoing segmental colectomy, the ERAS programme enabled earlier recovery and consequently shorter LOS [22-25]. Furthermore, a reduction of post-operative morbidity in patients undergoing intestinal resection was reported [26-29]. These results stimulated liver surgeons of the ERAS® group (Maastricht, Edinburgh and Tromsö) to adapt the ERAS programme to patients undergoing open liver resection. Van Dam *et al*. [30] found a significantly reduced LOS after open liver resection when patients were managed within a multimodal ERAS programme. Besides a reduction in median total LOS from 8 to 6 days (25%), the data also suggested that a further reduction in stay could be possible as there was a delay between the recovery and actual discharge of the patients [30]. Moreover, Stoot *et al*. found retrospectively that there was a further reduction in LOS from 7 days to 5 days when patients were operated laparoscopically and managed within an ERAS programme [31]. In that study there was also a delay between recovery and actual discharge of the patients. Previously, Maessen *et al*. reported a median delay to discharge of 2 days after patients had functionally recovered after colonic surgery managed within an ERAS programme [32]. This delay is often linked to patient age, hospital logistics, and absence of social and/or homecare support.

In most reported trials aiming at earlier recovery or a reduction in LOS, type of surgery and/or perioperative management were not standardised. In addition, the added value of laparoscopic LLS compared with open left lateral sectionectomy within an ERAS programme in terms of time to functional recovery, LOS in hospital, costs, and QOL has never been studied in a randomised controlled trial (RCT). However, randomisation of patients undergoing open or laparoscopic liver resection is hazardous. It is to be expected that experienced centres will be reluctant to randomise patients because of the absence of clinical and patient equipoise for laparoscopic resection. To capitalise on both centers with and without preference for laparoscopic liver surgery, and to thereby acquire an uninterrupted prospective series of patients, an alternative trial design with two randomisation arms (open versus laparoscopic surgery) and a prospective registry has been constructed for the ORANGE II trial. The combination of an RCT and a prospective registry will improve overall power and strengthen the external validity and generalisability of study results [33-35].

## Methods

### Ethics approval

The study has been approved by the Medical Ethical Review Board of the Maastricht University Medical Center, Maastricht, The Netherlands trial number NL 25591.068.08 / MEC 08-2-110. Ethics consent will also be obtained from the national or regional ethics boards in each participating country. Patients willing to participate in this trial will receive both verbal and written information at the time of recruitment in the outpatient clinic. In accordance with the local medical ethics committee all participating sites will provide an independent surgeon or physician if needed. An independent surgeon (M. Poeze) has been appointed for the Maastricht University Medical Center to answer questions. Confidentiality is guaranteed by assigning the participators an encoded trial number. This indicates that only the physician with the decoding ‘key’ will know which code number has been assigned to any patient. All trial data will be saved during the trial and stored on a server, and patients will be asked to consent to future analysis of these data. Withdrawal from the trial at any time or for any reason will not hold any form of consequences for the patient, and data from these patients will be deleted.

### Study design

The ORANGE II trial is a prospective superiority study with an experimental design, using two double-blinded randomised controlled arms and a prospective registry to determine whether laparoscopic surgery is to be preferred over open surgery in patients undergoing a LLS and participating in an enhanced recovery programme. In the participating randomising centers, patients, nurses and the ward physician (but not the operating surgeon) will be blinded for the type of intervention up to and including postoperative day (POD) 3. They will record the functional recovery criteria twice daily. Only the investigator and operating surgeons will know the actual procedure. The blinded ward physician(s) will decide on whether a patient will be discharged or not.

However, randomisation of patients undergoing open or laparoscopic liver resection is hazardous as previously explained. Moreover, another potential source of bias exists when randomising patients with a strong treatment preference. When patients cannot be blinded to their treatment allocation (POD 3) they may be resentful and demoralised if they do not receive their preferred treatment, and consequently they may have poor compliance. By contrast, patients receiving their preferred treatment may have above-average compliance.

Thus to capitalise on centres both with and without preference for laparoscopic liver surgery, and thereby to acquire an uninterrupted prospective series of patients, all non-randomised patients undergoing a LLS will be approached to participate in the prospective registry. Registration of these patients is imperative to guarantee a consecutive series of patients and also because the absence of such a series may restrict generalisation of the results, as randomised participants may not in fact be representative [36]. The combination of an RCT and a prospective registry will improve overall power and strengthen the external validity and generalisability of study results [33-35]. This non-randomised registry group will be analysed for centre and centre by treatment interaction as an observational study. Medical centres that wish to participate in this trial, but with liver surgeons early in the laparoscopic learning curve, will be accompanied during the procedure by an experienced proctoring laparoscopic HPB-surgeon.

### Primary & secondary endpoints

The primary endpoint of the ORANGE II trial is time to functional recovery. A patient is fully functionally recovered when all of the following five criteria are satisfied: 1) adequate pain control with oral analgesia; 2) restoration of mobility to an independent level; 3) absence of intravenous fluid administration; 4) ability to eat solid foods; and normal or decreasing serum bilirubin level and international normalised ratio.

It is medically justified to discharge patients when the criteria for full functional recovery are met and if the patient is willing to go home. Secondary endpoints include postoperative LOS in hospital, percentage of readmissions, total morbidity (both general and procedure related), composite endpoint of liver-surgery-specific morbidity, QOL, body image and cosmesis, reasons for delay of discharge after functional recovery, hospital and societal costs over 1 year, and long-term incidence of incisional hernias.

#### Morbidity

The preoperative morbidity status of patients will be measured using the American Society of Anesthesiologists (ASA) scale. The Portsmouth modification of the Physiological and Operative Severity Score for the Enumeration of Mortality and Morbidity (P-POSSUM) will be used to evaluate the risk of perioperative morbidity and mortality. Post-operative morbidity is rationally predictable, with hemorrhagic complications occurring predominantly during surgery or in the early postoperative phase, and biliary complications, intra-abdominal abscess, or liver failure in the later postoperative phase. Wound infection and sepsis will be additional complications that require monitoring. Morbidity will be classified and analysed according to the validated classification for postoperative morbidity as described by Dindo *et al*[37].

#### Liver resection-specific composite endpoint

In this trial, we will also use a well-defined liver surgery-specific composite endpoint, as suggested by van den Broek *et al*. [38]. This endpoint is a parameter composed of a combination of procedure-specific complications, which is considered as a single, dichotomous outcome: operative mortality, intra-abdominal haemorrhage, ascites, bile leakage, intra-abdominal abscess, and post-resectional liver failure. These components, which are all specific to liver surgery and have substantial clinical relevance, reflect complications rated as Dindo grade 3–5. A composite score of 1 (failure) will reflect the occurrence of at least one of the above liver-specific complications, and a score of 0 (success) will be assigned if none of these occur.

#### Quality of life

To assess QOL in patients undergoing laparoscopic versus open LLS, the Dutch version of the EuroQol five-dimension (EQ-5D) status test in Dutch centers and the translated EQ-5D for international centers will be used. The EQ-5D is a standardised instrument for use as a measure of health outcome, which consists of the five dimensions of mobility, self-care, usual activities, pain/ discomfort, and anxiety/depression, with three levels each and a rating on the EQ visual analogue scale (VAS; 0–100). [39-41] Furthermore, the European Organisation for Research and Treatment (EORTC) 30-item post-cancer QOL questionnaire (QLQ-C30; with the liver metastases (LM21) module will be used for liver-specific treatment measurements [42]. Assessment of the patients’ QOL will be performed at the time of consent, discharge and 10 days, 3, 6 and 12 months after discharge.

#### Body image and cosmesis

To evaluate differences in postoperative body image and cosmesis, the Body Image Questionnaire (BIQ) will be used [43,44], which consists of eight questions about body image and cosmesis. The body image assessment will be performed preoperatively at time of consent. Both the body image and cosmesis assessment will take place at discharge, and at 10 days, 3 months, 6 months and 12 months after discharge.

#### Hospital and societal costs

The economic evaluation will include a cost-utility analysis from a Dutch societal perspective. The incremental costs per quality adjusted life year (QALY) gained will be based on utility scores from the EQ-5D [39-41]. All hospital expenses (direct and indirect) related to both interventions will be monitored. In addition, a cost questionnaire offered at the regular follow-up consultation (3, 6 and 12 months) will help assess the societal and individual costs outside health care relating to patients’ absence, impaired mobility, work, or normal daily activities. Unit prices will be based either on prices from the participating hospital financial departments or will be extrapolated using Dutch guidelines for cost calculation [45].

#### Incidence of incisional hernias

Incisional hernia after open surgery is a well-known complication of surgery, with an incidence of up to 20% after a 10-year period [46]. In patients undergoing a sigmoid resection, Anderson *et al*. found that laparoscopic resection led to a significantly lower incidence of incisional hernia compared with open surgery [47]. Furthermore, in two retrospectively analysed series of patients who received a partial hepatectomy, different types of incisions were compared. D’Angelica *et al*. reported that the common incisions used for partial hepatectomy were the Mercedes incision and extended right subcostal (ERSC) incision, and that the ERSC incision provides adequate, safe access and is associated with fewer long-term wound complications (9.8% vs 4.8%, *P* ≪ 0.001) [48]. More recently, Togo *et al*. reported frequencies of incisional hernia after median, J-shaped, right transverse incision with a vertical extension at the midline from the subumbilical region to the xiphoid process (RTVE), and reversed T incisions to be 6.3%, 4.7%, 5.4%, and 21.7%, respectively. A diagnosis of ‘no hernia’ required a minimum follow-up of 12 months [49].

To assess the incidence of incisional hernias in patients undergoing laparoscopic and open LLS, they will be contacted at a mean time of 1 year after resection to undergo ultrasonography to assess the incidence of incisional hernia.

### Study population

**Figure 1:**
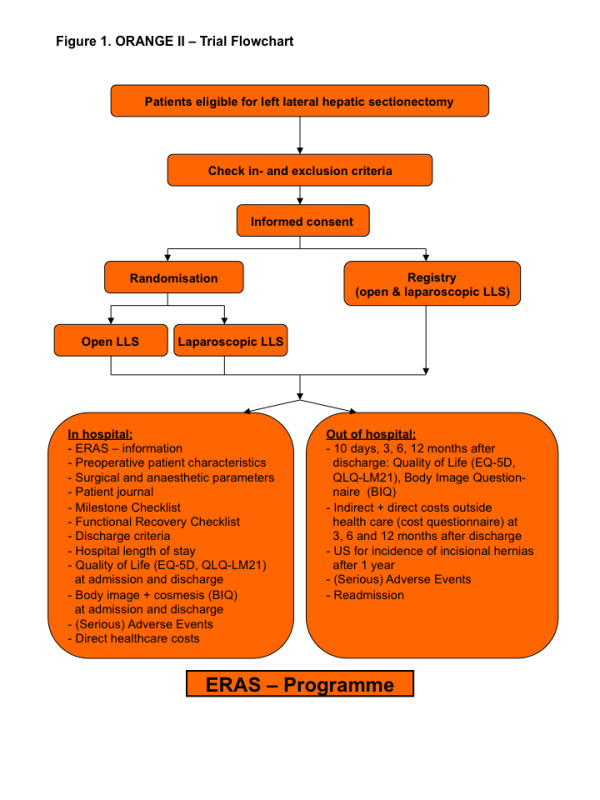
Orange II-Trial Flowchart.

Every patient requiring an LLS will be identified and informed at the outpatient clinic about open and laparoscopic liver resection. Only patients meeting the inclusion and exclusion criteria will be approached for randomisation. After reading the ORANGE II trial patient information and being allowed 1 week for consideration, patients will be asked for their informed consent. All patients ineligible for randomisation will be approached for participation in the prospective registry. If patients express an explicit preference, they will be allocated to the prospective registry and interviewed to ascertain the reasons for their preferences. Personal written informed consent will be obtained for all groups. Randomisation will be carried out through the ORANGE II trial website using web-based randomisation software (TENALEA®; www.tenalea.com) (see Figure 1 for trial flow-chart).

Patients will be approached for randomised inclusion if they meet each of the following inclusion criteria: require LLS; willingness to participate in the study; able to understand the nature of the study and what will be required of them; are men or non-pregnant, non-lactating women between the ages of 18 and 80 years of age; have a body mass index of between 18 and 35; and have ASA grading of I to III.

The exclusion criteria are: liver resection other than LLS; underlying liver disease; unwillingness to participate; inability to give written informed consent; and ASA grading of IV to V.

### ERAS-programme

**Figure 2:**
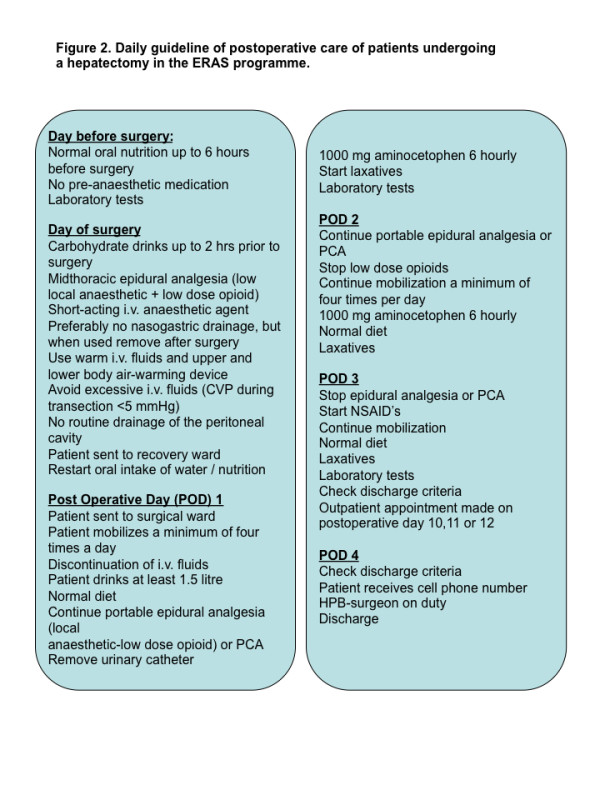
Daily guideline of postoperative care of patients undergoing a hepatectomy in the ERAS programme.

All patients will participate in the ERAS liver programme,with a standardised peri-operative management. For daily guidelines of the pre- and postoperative care of patients undergoing liver resection (Figure 2).

### Functional recovery criteria

The evaluation of time to functional recovery will start on POD 0 and will be scored twice daily until discharge from the hospital. The discharge process starts at the pre-admission counseling session, during which any special needs of the patients will be determined (for example, homecare or social support, transport. Before admittance, any problem that could delay discharge will be identified and addressed. Patients will only be discharged when they have met the functional recovery criteria and are willing to go home. Reasons to delay discharge after functional recovery will be monitored and documented. Functional recovery criteria and LOS in hospital will be independently monitored and analysed.

#### Adequate pain control with oral analgesics

Postoperative pain will be systematically registered twice daily using the validated verbally administered 11-point numeric rating scale (NRS-11, 0 to 10) [50-53]. Members of a specialised pain team will ask patients to rate the intensity of their current pain on a scale of 0 (no pain) to 10 (worst possible pain), with pain rated as ‘mild’ (1 to 3) ‘moderate’ (4 to 6) or ‘severe’) 7 to 10 [54]. The NRS-11 seems to be better accepted by most patients and to be at least as sensitive and valid as the more traditional VAS ratings [53].

#### Tolerance of solid food

Fluid and solid food intake will be monitored and must return to normal, that is, when oral intake of water or normal food is resumed and continued for at least 24 hours. Furthermore the incidence of postoperative nausea and vomiting, which obviously influences intake, will be monitored postoperatively until day 6 using a scale ranging from 0 (no nausea) to 10 (worst possible nausea), and where necessary, be countered prophylactically by antiemetic treatment.

#### Mobility

To assess the difference between the preoperative and postoperative mobility level, the ERAS Mobility Scale (EMS) has been developed from the Groningen Activity Restriction Scale [55]. The EMS assesses 10 basic actions to compare the level of mobility before and after surgical intervention. When the patient is able to perform 8 of the 10 items, they are independently mobile. Patients will be assessed whether they are able or not to independently perform these basic actions fully. Daily the assessment will be repeated and compared with the preoperative baseline score until mobility at an independent or preoperative level is achieved.

### Statistical analysis

#### Sample size

Because laparoscopic liver surgery focuses on accelerated recovery, time to functional recovery is used as the primary outcome parameter. Owing to the lack of hard evidence about the reduction in time to functional recovery after liver surgery, we have chosen to use the parameter that most accurately approaches our primary endpoint for our power calculation (LOS). Based on a retrospective analysis of 31 patients in both ERAS and non-ERAS settings, who have undergone LLS from 1990 to the present time, the mean ± SD post-operative hospital stay for a LLS in the Maastricht University Medical Center is 6 ± 2.73. It therefore seems that that in patients undergoing laparoscopic LLS, time to functional recovery is reduced in comparison to patients undergoing the open procedure. We are aiming for a reduction in time to functional recovery of 2 days. A sample size of 2 × 40 patients in the randomisation arms will be sufficient to show a 2-day reduction with a power of 90% and a level of significance at α = 0.05 (two-tailed, given a within-arm SD of 2.73 with effect size *d* = 0.73). Assuming an expected withdrawal rate of ≤ 10% during the trial, the participation of at least 10 centres, and the required addition of one randomised patient per arm for every additional participating centre (C) to compensate for the loss of degrees of freedom incurred in the data analysis, which takes centre and treatment × centre effects into account, a total sample size of 110 (n = 2 × 55) will be required.

For all secondary outcome measures, the power will be 75% after correction for multiple testing with two-tailed α = 0.01, assuming the same effect size (*d* = 0.73) as for the primary outcome. An interim analysis of the primary outcome, using Snapinn’s method, will be performed after inclusion of 50% of the sample to avoid unnecessary inclusion of too many patients in this ORANGE II trial [56].

#### Descriptive statistics

The primary outcome parameter of time to functional recovery and the secondary parameter of LOS in hospital will be given in days, with a median and range. Morbidity will be classified according to the classification described by Dindo *et al*. and defined as a dichotomous composite endpoint, while readmission will be given as a percentage. Scores for quality of life, body image and cosmesis will be given as mean and standard deviation per time point per treatment arm. Hospital costs will be given as median and range. Long-term incidence of incisional hernia will be reported and analysed.

#### Univariate analysis

The primary outcome measure of time to functional recovery will be measured in days, and will be analysed with fixed-effect regression that will take centre and treatment × centre interaction into account as fixed effects. If the actual number of centers and the sample size per centre allow random effects analysis, this will also be performed and this analysis will have the same power as the planned fixed effects analysis if the design effect does not exceed 1.2. With a sample size of 10 patients per centre, the design effect is 1.2 if the intraclass correlation (ICC) is 0.02, where the ICC is based on treatment × centre interaction [57].

All secondary outcomes as measured at discharge will be analysed by fixed-effect regression using linear regression for quantitative outcomes and logistic regression for binary outcomes, and including the baseline measure as a covariate to improve power and precision. In addition to *P*-values, confidence intervals for all effects will be reported. Morbidity will be classified as described by Dindo *et al*., but will be presented as raw data only because the required sample size for intervention effects on morbidity is much larger than the calculated sample size for this trial [58].

#### Economic evaluation

The economic evaluation will include a cost-utility analysis from a societal perspective. The time horizon of this evaluation will be the same as the duration of the trial, that is, 12 months. All costs (direct and indirect) related to both interventions will be calculated. The final cost calculation of unit costs will be based on a combined bottom-up and top-down approach. In accordance with Dutch guidelines for cost calculation, indirect healthcare costs will not be taken into account. In addition, resource use will be measured by use of primary data that is registered in our case record forms (CRFs) by use simple checklists. Furthermore, a questionnaire will be used to survey the direct non-healthcare costs related to travelling, impaired mobility and domiciliary care (for normal daily activities). The incremental, indirect non-healthcare costs per QALY gained will be based on the utility scores from the EQ-5D [39-41]. For all direct healthcare costs, the unit prices will be based either on prices from the hospital financial department or the Dutch guidelines for cost calculation [45].

#### Registry

The prospective registry of patients who cannot be randomised because of ineligibility or because of explicit treatment preference on the part of the patient or surgeon will be analyzed as an observational study. In addition, data from the registry will be analyzed for interaction between treatment, centre, and study type (randomised or not). On condition that there is no interaction between treatment, centre, and study type, and that the observational study does not suffer from severe confounding (because adjusting for that strongly reduces the power of the observational study), pooling of both studies should give more power than separate analyses of either study. Possible confounders will be registered in the CRFs. The inclusion of the prospective registry in the trial design will create an uninterrupted case series, which will increase external validity and generalisability.

#### Data collection

Data concerning patient characteristics, functional recovery, surgical and anaesthesiologic parameters, morbidity, LOS, QOL, patient compliance, and costs will be prospectively collected using both paper CRFs and an open source clinical trial software platform (OpenClinica®; Ikaza Research, Cambridge, MA, USA) that uses e-CRFs for electronic data capture and clinical data management, which are validated and stored in compliance with good clinical practice guidelines. The e-CRFs will be stored in a secured database (Oracle Cor., Redwood Shores, CA, USA), and as stated previously, all patient data will be encoded to ensure privacy.

#### Monitoring

For this trial, a Data and Safety Monitoring Board (DSMB) has been appointed that will consist of three members: a chairperson, an independent statistician, and a medical specialist. In a concerted effort a DSMB charter will be developed, and all three members will sign a non-competing interest form. The DSMB will be responsible for safeguarding the interests of trial participants, assessing the safety and efficacy of the interventions during the trial, and monitoring the overall conduct of the clinical trial.

#### Intention to treat

Analysis of all patients will be performed according to the intention-to-treat principle: patients will be analysed as randomised or as planned in the non-randomised prospective registry, and all patients will be included in the data analysis with proper methods for handling missing data.

## Discussion

Several authors have indicated that laparoscopic liver resection has many benefits over conventional open liver resection. However, this has never been proven in an RCT, and what the primary endpoint should be for an RCT comparing open and laparoscopic liver resection is a subject to debate. Using either liver surgery-related mortality or liver surgery-specific morbidity as an endpoint is not feasible, because patient accrual would take many years and be a logistically major global effort [58]. LOS in hospital, time to recovery, long-term incisional hernias, body image, and costs are potential candidates because improvements in these are some of the possible benefits. Laparoscopic liver resection is appealing for many surgeons and patients, but the learning curve for the surgeon is thought to be long and costly for hospital budgets. However, operating times in laparoscopic LLS tend to be shorter, and may compensate for expenses in technology and consumables [31,59]. Moreover, the existing trials in liver surgery have not evaluated time to recovery or LOS in hospital after laparoscopic liver resection within an enhanced recovery programme. The more rapid recovery reported after enhanced recovery programmes may be further accelerated as a consequence of small incisions in laparoscopic surgery. In addition, learning curves for laparoscopic left lateral resection or anterior segments seem to be reasonably short for liver surgeons with advanced laparoscopic experience [60]. The question remains whether an RCT is necessary to prove that laparoscopy should be accepted as the preferred method to perform liver resection. In the Louisville consensus meeting on laparoscopic liver surgery, it was stated that laparoscopic LLS should be standard practice in experienced hands [61]. However, this may have been a subjective vision of a subset of opinion leaders, because long experience with both open and laparoscopic liver surgery was the main characteristic of those attending the meeting. Undoubtedly, the dissemination phase of laparoscopic liver surgery has started, and it is to be expected that many surgeons will adopt this technique in the future. A multinational multicentre prospective registry, a well-organised multicentre RCT, training programmes, and quality control measures are of great importance during this adoption period [33].

It is well recognised that a well-conducted double-blind RCT provides the highest level of evidence to prove the possible benefits of laparoscopic liver resection. However, performing an RCT in surgery is not without difficulties, and alternative trial designs may be necessary [33,34,62]. First, the intervention needs to be tested in a standardised environment, and the properties of the intervention should remain unchanged during the trial period. This seems impossible for an intervention such as laparoscopic liver surgery in a multicentre RCT. Experience varies between participating centres, and will vary over time. Moreover, local standards for perioperative care are different. Both LLS and the ERAS® enhanced recovery protocol provide the standardisation needed. The learning curve of a LLS is short in centres with experience in liver surgery and advanced laparoscopy. The use of proctor surgeons in centres with limited experience in laparoscopic liver surgery the operative techniques can be reasonably standardised, and this should eliminate learning curve influences on outcome parameters. Quality of the surgery can be assured by digital video recording.

Second, the intervention should be double-blinded. Although double blinding in a surgical trial is difficult, using a fixed abdominal dressing for 3 days after surgery is feasible, and should prevent both ward caregivers and patients from knowing the type of intervention.

Third, it is reasonable to query whether this is now the right time to perform an RCT and whether the results of the trial will be valid for the more general surgical community. A recent review of the results of laparoscopic liver resection in 2,804 patients showed that laparoscopic liver resection in expert centres is feasible and safe for both minor and major liver resections [36]. The percentages of liver resections performed laparoscopically now range from 25% to 65% in high-volume expert centres such as University Hospital Southampton NHS (Southampton, UK), Henri Mondor (Paris, France), UPMC (Pittsburgh, USA), UZ Leuven (Leuven, Belgium) and Rikshospitalet (Oslo, Norway) [36,63]. Although it is to be expected that many centres worldwide will adopt laparoscopic liver resection as a more or less standard procedure in the near future, there are still many patients and surgeons that prefer the open procedure long beyond the learning curve. In parallel with the development of laparoscopic liver surgery, ‘fast-track’ programmes in various areas of surgery, including liver surgery, are gaining popularity. Therefore, this seems to be the right time for this RCT to be performed. The multicentre character of the ORANGE II trial with randomisation of patients and surgeons with treatment equipoise and a prospective registry to cover both surgeons who believe that based on their laparoscopic experience randomisation is not ethically justified and patients with a strong treatment preference will provide external validity. This trial design capitalises on rather than ignores the differences between patients, will provide more robust outcome data, and should lead to continuous performance monitoring after the trial [35,62].

The key question clearly is as to whether this RCT is really necessary. The benefits of laparoscopic liver resection are not beyond reasonable doubt, and although data are becoming increasingly available, recent publications do not provide sound data on time to recovery. Worldwide, median LOS in hospital for open and laparoscopic resections varies from 4 to 8 days [17,30,64-66]. Reasons for delay in discharge and discharge location are often absent, and a clear definition of recovery has not been used to date in any of the publications. Departing from the standpoint that an RCT should be conducted, the question is which sample size should be used? In our opinion, a reduction of only 1 day in time to recovery or LOS in hospital after laparoscopic resection would be a disappointingly low gain. To prove such a reduction, 320 patients would be needed (α = 0.05 and power of 90%), making the trial unlikely to be accomplished. Based on available reports, a 2-day reduction should be possible [17,31,66], and reduces the sample size to 110 patients undergoing LLS. This number is reasonably moderate, and it is to be expected that patient accrual will be accomplished within 1–2 years.

It should be realised that many centres have introduced laparoscopic liver surgery programmes in the absence of a central reporting or certifying agency. In our opinion, laparoscopic LLS should function as a model for further dissemination of laparoscopic techniques in hepatic surgery. The left lateral segment of the liver has been a natural first step for a laparoscopic resection given the peripheral anatomical location (thin liver segment, minimal requirement for biliary dissection, and ease of controlling the left portal pedicles and left hepatic vein), and has been proven to be safe and feasible with reproducible results [20,36]. The implementation of the laparoscopic LLS may not only serve as a guide to develop and master programmes for major laparoscopic hepatic resections, but may also be used as an introduction for centres new to laparoscopic approaches in liver surgery. To adopt laparoscopic liver resection safely, certification for centres, surgeons, and units should be available through the International Hepatobiliary (HPB) Association, and national and international HPB associations should become involved in the goal of establishing training standards and credentials to ensure a high and consistent outcome. The ORANGE II trial in which techniques are standardised and a training and proctor programme is available, combined with the hybrid design of randomisation and registry may help to provide a framework for controlled and safe implementation of laparoscopic liver resection across participating centres.

## Conclusions

The international multicentre randomised controlled ORANGE II trial is based on the observations of more rapid recovery and discharge after laparoscopic liver resection, and more rapid recovery and discharge after open liver resection within an enhanced recovery programme. This is the first RCT to provide evidence on the merits of laparoscopic surgery in patients undergoing a LLS within an enhanced recovery programme.

## Trial status

Currently there are nine actively participating centres, consisting of the Maastricht University Medical Center (MUMC), Academic Medical Center (AMC), University Medical Center Utrecht (UMCU), Erasmus Medical Center (Erasmus MC), University Medical Center Groningen (UMCG), Maxima Medical Center (MMC), Medical Spectrum Twente (MST) in The Netherlands. In addition, the University Hospital Aachen (UK Aachen) and San Raffaele Hospital Milan (HSR Milan) are two participating centres in Germany and Italy. Additional centres will be invited to participate.

## Abbreviations

ORANGE = Optimised recovery with accelerated nutrition and GI enhancement; ERAS = Enhanced recovery after surgery; LLS = Left lateral sectionectomy; LOS = Hospital length of stay; POD = Post operative day; CRF = Case record form; ASA = American society of anaesthesiology; P-POSSUM = Portsmouth physiologic and operative severity score for the enUmeration of mortality and morbidity; EORTC = European organization for research and treatment of cancer.

## Competing interests

Both positive and negative results will be published. There are no financial interests in this trial and none of the parties concerned has right of veto.

## Author’s contributions

RMvD, EMW-L-H and JRvdV drafted the manuscript. RMvD, EMW-L-H, JRvdV, JHMBS, MHAB, SWMOD, KL, and CHCD participated in the design of the study and revised the manuscript. GJPvB performed the sample size calculations, advised in trial design, leads interim analysis and revised the manuscript. All authors read and approved the final manuscript.
